# FINCA – a conceptual framework to improve interprofessional collaboration in health education and care

**DOI:** 10.3389/fmed.2023.1213300

**Published:** 2023-10-02

**Authors:** Matthias J. Witti, Jan M. Zottmann, Birgit Wershofen, Jill E. Thistlethwaite, Frank Fischer, Martin R. Fischer

**Affiliations:** ^1^Institute of Medical Education, LMU University Hospital, LMU Munich, Munich, Germany; ^2^Faculty of Health, University of Technology Sydney, Ultimo, NSW, Australia; ^3^Chair of Education and Educational Psychology, Department of Psychology, LMU Munich, Munich, Germany

**Keywords:** interprofessional education, interprofessional collaboration, collaborative problem-solving skills, observable collaborative activities, learning, teaching, scaffolding

## Abstract

The health care system in Germany and in many other countries is facing fundamental challenges due to demographic change, which require new integrated care concepts and a revision of the collaboration between health care professions in everyday clinical practice. Internationally, several competency framework models have been proposed, but a framework that explicitly conceptualizes collaborative activities to improve interprofessional problem-solving competency in health care is still missing. Such a framework should define contextual, person-related, process-related, and outcome-related variables relevant to interprofessional problem solving in health care. Against this background, we present a conceptual framework to improve interprofessional collaboration in health education and care (FINCA) developed with scientific consideration of empirical data and various theoretical references. FINCA reflects an interprofessional learning and interaction process involving two persons from different health care professions and with different individual learning prerequisites. These two initially identify a problem that is likely to require interprofessional collaboration at some point. FINCA acknowledges the context of interprofessional learning, teaching, and working as well as its action-modifying context factors. We follow the reasoning that individual learning prerequisites interact with the teaching context during learning activities. At the heart of FINCA are observable collaborative activities (information sharing and grounding; negotiating; regulating; executing interprofessional activities; maintaining communication) that can be used to assess individuals’ cognitive and social skills. Eventually, the framework envisages an assessment of the outcomes of interprofessional education and collaboration. The proposed conceptual framework provides the basis for analysis and empirical testing of the components and variables it describes and their interactions across studies, educational interventions, and action-modifying contexts. FINCA further provides the basis for fostering the teaching and learning of interprofessional problem-solving skills in various health care settings. It can support faculty and curriculum developers to systematize the implementation and improvement of interprofessional teaching and learning opportunities. From a practical perspective, FINCA can help to better align curricula for different health professions in the future. In principle, we also see potential for transferability of the framework to other areas where different professions collaborate.

## 1. Introduction

The health care system in Germany, as well as in many other countries, is facing fundamental challenges due to demographic change. With the aging of the world’s population, on the one hand, the care of the elderly is gaining in importance – on the other hand, the associated need for treatment and management of complex chronic long-term conditions is becoming increasingly important (1, 2). These changes require new integrated care concepts and a revision of the cooperation between health care professions in everyday clinical practice. Numerous position papers and strategic plans have therefore been calling for an increased integration of interprofessional education in under-and postgraduate training in medicine as well as other health care professions (3–7).

Interprofessional collaboration (IPC) involves different health and social care professions meeting regularly to negotiate and agree on how to solve complex care problems or deliver services. Interprofessional teamwork is characterized by a high level of team identification and close networking and interdependence. The dimensions of IPC also include clear team goals, a shared team identity, shared team commitment, and clear role allocation (8). Interprofessional education (IPE), which is necessary to prepare for IPC, takes place whenever trainees, students, or professionals from two or more professions come together to learn with, from, and about each other in order to optimize collaboration in patient care (9). Both definitions reflect the need for IPE and IPC to include interactive problem-solving processes and related activities.

Similarily to other educational interventions, it remains challenging to demonstrate a causal relationship between IPE and general care system outcomes (such as improved clinical experience or improved patient experience). Multiple studies have demonstrated that IPE interventions can lead to improved patient care in specific contexts [e.g., (10, 11)]. It must be noted, however, that due to the heterogeneous nature of these studies and the variety of IPE interventions, it is rather difficult to integrate and generalize results of these interventions to inform general theory-building (12).

Several competency framework models in IPE have been proposed internationally, primarily motivated by health policy makers [e.g., (13–16)]. These framework models address both IPE and IPC and formulate overarching competency goals for successful interprofessional work. The frameworks include ethics and values, teamwork, leadership, conflict resolution, communication, mutual respect, role clarity and patient-centredness as important areas (17).

As the need for IPE and IPC increases due to the demands brought about by demographic change, we believe there is also a growing need for a framework that explicitly conceptualizes collaborative activities to improve interprofessional problem-solving skills in health care. Such a framework should define contextual, person-related, process-related, and outcome-related variables relevant for interprofessional problem-solving in health care. At the same time, it should be based on observable activities that allow for an operationalization of interprofessional problem-solving skills.

In this paper, we propose a framework based on a combination of three theoretical strands from educational psychology research on collaborative learning: (1) fostering of diagnostic competencies (18), (2) collaboration scripts (19), and (3) collaborative problem-solving skills (20). These theoretical strands have proven useful in different contexts and domains such as teacher and medical education (see Section 2.2). To our knowledge, these generalizable educational frameworks and theories have not yet been utilized to inform and enrich the development and design of competency framework models for improved IPE and IPC.

On this basis, we offer definitions and operationalizations that will enable empirical research studies to assess and subsequently foster collaborative problem-solving skills, as well as the integration of results across diverse IPE and IPC contexts. Quantitative methods could thus be increasingly employed in the study of IPC, which to date has been primarily of a qualitative nature (21). Moreover, such a framework could serve as an educational tool by providing the foundation for fostering the teaching and learning of interprofessional problem-solving skills in various health care settings.

## 2. Developing a conceptual framework for analyzing and fostering interprofessional problem-solving skills

### 2.1. Development context

The conceptual **F**ramework to **I**mprove i**N**terprofessional **C**ollaboration in health education and c**A**re (FINCA) presented in this article was developed by the authors in the context of the Graduate School “Interprofessional Teaching in the Health Professions” (ILEGRA) which was funded by the Robert Bosch Stiftung from 2018 until 2022. ILEGRA served to promote young scientists and was conducted in cooperation between the University of Osnabrück and LMU Munich. ILEGRA research fellows came from a variety of health care professions and worked on dissertation topics related to teaching, assessing and evaluating in the context of IPE or health care practice. ILEGRA brought together researchers from different disciplines to serve as scientific supervisors or advisory board members, some of whom work outside the health professions. This allowed for a broad exchange of experts from the health professions with experts from educational psychology, adult education, work and organizational psychology, and sociology. The approach resembled focus group discussions (22) and took into account existing framework concepts and the variables they contain. The iterative discussion rounds with all experts and with the ILEGRA fellows informed the development and conceptual design of the present framework, offering new perspectives beyond the health professions.

### 2.2. Theoretical sources beyond IPE and IPC to inform the development process

The proposed framework addresses all educational scientists and curriculum developers in the field of IPE to contribute to better IPC processes and outcomes. The development of FINCA was guided by three theoretical strands beyond the aforeementioned competency framework models for IPE and IPC:

(1) The first strand is an *interdisciplinary framework on the acquisition and fostering of diagnostic competencies* by Heitzmann et al. (18) with its basic assumption that one’s own cognitive activities are an important prerequisite for the acquisition of competencies. Disciplines are defined as broad academic fields, such as anthropology, economics and geography (8). We propose to conceptualize collaborative activities for interprofessional problem-solving on the basis of this framework which also considers a wide range of individual prerequisites (cognitive professional abilities as well as motivational and affective factors) and also context factors that could potentially moderate collaborative activities. FINCA was further inspired by Biggs’ 3P model (Presage, Process, and Product) of teaching that shows how learner prerequisites interact with the teaching context during learning activities and relates them with learning outcomes (23).
(2) The second strand assumes that the extent to which IPC takes place in specific situations depends on the thought processes and activities of the individuals involved, drawing on cognitive structures like *illness scripts* and *collaboration scripts*. Illness scripts were first used to explain the diagnostic behavior of physicians. However, their usefulness is also being advocated in the nursing context [e.g., (24)]. In short, medical or nursing knowledge is organized into illness scripts which consist of patterns for diseases or clinical dysfunctions, their underlying pathophysiological processes and symptoms, as well as their care courses including therapeutic interventions (25). With clinical experience, these illness scripts develop further as increasingly efficient ways of thinking and work organization of physicians, nurses, and allied health professionals to solve and manage clinical problems. Thus, the continuous development of illness scripts enable health professionals to speed up and improve the quality of their decisions based on recurrent patterns.

Particularly in the educational context, collaboration scripts are also described in the literature (19). *Internal collaboration scripts* can be understood as a person’s current knowledge of implicit and explicit rules for effective and efficient collaboration. *External collaboration scripts* can, in turn, be understood as sets of scaffolds that help to structure collaborative learning processes. They may gradually become internalized as learners act in accordance with the script content (26). While only few empirical studies are available to date regarding the use of collaboration scripts in a medical context [e.g., (27, 28)], the consideration of internal collaboration scripts of health professionals in the context of IPE and IPC is promising to support the development and application of collaboration knowledge.

(3) The third strand encompasses *collaborative problem-solving skills* that are crucial when two or more health professionals interact and orchestrate knowledge and skills to solve a shared problem. Interprofessional interactions are characterized by a diversity of professional backgrounds, distribution of responsibilities, and different approaches and values with regard to the provision of care. Interprofessional collaborative practice is dependent on the competencies of each professional group to ensure optimal care for patients, families and communities. In such situations, competencies must be integrated and a common level of information must be established, which requires a high degree of collaborative problem-solving competence from all professionals involved. Liu et al. (20) studied collaborative problem-solving in groups, describing social skills such as sharing ideas, negotiating ideas, regulating problem-solving activities, and maintaining communication. Following these considerations, we consider collaborative problem-solving skills as indispensable prerequisites for a person to participate effectively in a process in which two or more participants attempt to solve a problem together.

## 3. Structural components of FINCA

**Figure 1:**
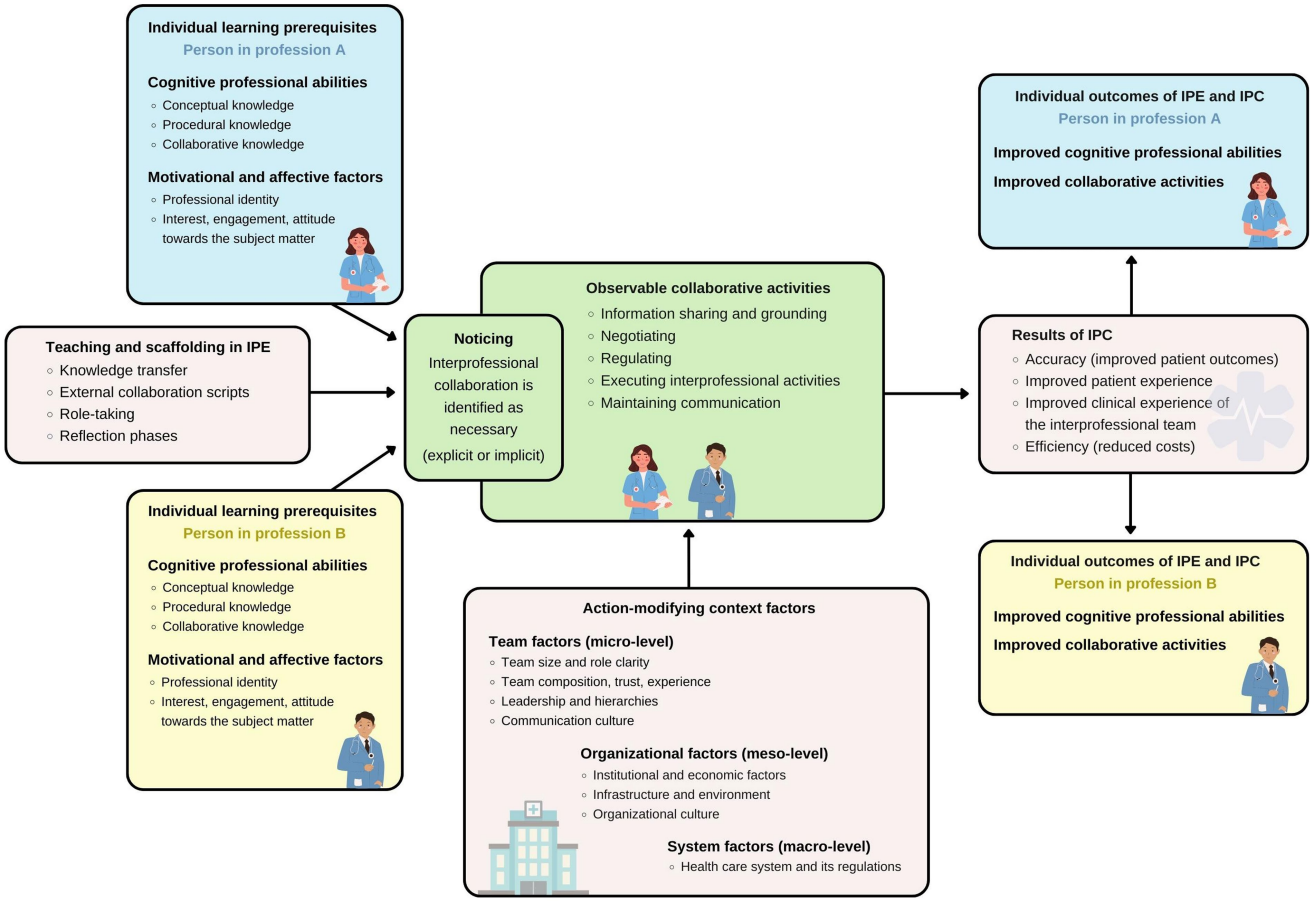
The conceptual framework to improve interprofessional collaboration in health education and care (FINCA). Arrows visualize the influence of the respective activities and factors. For instance, teaching and scaffolding in interprofessional education (IPE) affects noticing and the partially overlapping observable collaborative activities. Both individual learning prerequisites of the persons from different professions involved in the interprofessional collaboration (IPC) and action-modifying context factors influence these collaborative activities. Eventually, results of IPC arise from the collaboration and individual outcomes of IPE and IPC can be assessed for the persons involved.

FINCA (see Figure 1) reflects an interprofessional learning and interaction process that involves two different health care professionals (i.e., person in profession A and person in profession B) with different *individual learning prerequisites* (18). These two persons recognize a problem that presumably requires interprofessional collaboration at one point – we term this *noticing*, which involves recognition and identification, but can be explicit or implicit/tacit (29, 30). FINCA further acknowledges the context of interprofessional learning, teaching, and working and its *action-modifying context factors*. We follow the reasoning that individual learning prerequisites interact with the teaching context during learning activities (18, 23). At the core of FINCA are *observable collaborative activities* [*cf.* (20)]. At the same time, the framework envisages an assessment of the *outcomes of IPE and IPC*. In the following sections, the core content aspects of the framework are explained and pragmatic research approaches will be outlined.

### 3.1. Teaching and scaffolding in IPE

Learners and practitioners across all health professions collect and evaluate multiple pieces of clinical information to make decisions about patient care. In doing so, both learners and practitioners use an analytical approach called clinical reasoning. Clinical reasoning refers to all cognitive processes underlying these decisions (31) and includes medical problem-solving and medical decision-making.

In an interprofessional context, collaborative clinical reasoning can lead to a shared mental model about patient problems and further care (32, 33). This includes an interprofessional comparison of different diagnoses or dysfunctions and the process of care, patient monitoring, explanation of treatment options, and team communication. Visser et al. (34) conclude that learners from different health professions discussing treatment plans together would benefit more in their learning process. This is because learners would have to (a) structure their thoughts (cognitive level) and (b) have to provide explanations for learners from other professions, answer their questions, and give feedback to them (metacognitive level) in order to create a common knowledge base.

Both educational research in general and research in health professionals’ education in particular have shown that additional instructional support is needed for learning from challenging problems in complex learning scenarios (18, 28, 35, 36). FINCA therefore suggests various support measures in the context of teaching and learning as important variables that may influence or moderate the observable collaborative activities.

Central to *scaffolding* is supporting learners by directing attention while they work on a task. This can, for instance, be done by providing cues, case illustrations, or prompts. Numerous empirical studies have demonstrated the effectiveness of scaffolding for *knowledge transfer* [see (35)]. A wide variety of types of socio-cognitive scaffolding have been developed for collaborative problem-solving scenarios, e.g., in the form of *external collaboration scripts* (19). Instructional support with external collaboration scripts can foster learning processes by introducing a sequence for collaborative activities. External collaboration scripts can (a) specify certain activities to be performed by learners, (b) predetermine the timing of activities, or (c) specify collaboration roles and interaction activities (37). *External collaboration scripts* have been developed for both face-to-face and computer-mediated settings and have been largely successful in improving collaboration processes as well as individual learning outcomes (38). In addition, scripts promote collaborative activities such as exchanging new ideas, asking questions, or negotiating between learning partners. Furthermore, collaboration scripts support deeper cognitive elaboration in individual learners. The use of collaboration scripts such as the handover tool SBAR (Situation, Background, Assessement, Recommendation) and its derivatives has shown promising results in mono-and interprofessional patient care [e.g., (39, 40)]. The SBAR tool is a scheme for structuring communication processes for the exchange of patient information (41) that organizes this information, reminds of important content and details that may otherwise be lost, and reduces leaps of thought and omissions which is critical to patient safety in a complex system (42, 43). The use of SBAR has even been recommended by the WHO (44).

Another promising type of scaffolding for IPE could be provided by assigning specific roles to learners to reduce the full complexity of a task. Through *role-taking*, the perspective on the full task can be focused on key learning points. In interprofessional encounters, the roles of the collaborating health professionals and the role of the patient are typical. Systematic role change allows for new perspectives and learning. Additionally, learners can be assigned the role of an observer. Results on acquiring diagnostic competences in the role of an observer are still lacking, but Stegmann et al. (45) showed that communication skills can be acquired effectively in this role.

*Reflection phases* are another scaffolding approach that holds potential to foster IPE. Nguyen et al. (46) provided a comprehensive definition of reflection as “the process of engaging the self in attentive, critical, exploratory and iterative interactions with one’s thoughts and actions, and their underlying conceptual frame, with a view to changing them and with a view on the change itself” (p. 1182). Guided reflection can take place before, during, or after an event that requires IPC. Different types of guided reflection have been reported to efficiently foster the acquisition of diagnostic competences in medicine (47, 48). There are three main reasons why reflection could be beneficial for learning: (1) Reflection phases add a pause that learners might use to better retrieve and apply conceptual knowledge with less time pressure. Learners might also use such a pause to evaluate the selected strategy and think about alternatives. (2) Learners may generate self-feedback to advance their learning during guided reflection. (3) Reflection phases may also support the planning of subsequent steps in the collaboration process.

To date, there has been little systematic research on what forms of scaffolding are suitable in IPE to foster interprofessional problem-solving skills (49). However, *learning with simulations* has been shown to have great potential in this regard (50, 51). Against this background, the following support measures have been included by way of example in FINCA, but this list can of course be extended: *knowledge transfer, external collaboration scripts, role-taking,* and *reflection phases*.

### 3.2. Individual learning prerequisites

In any interprofessional interaction, at least two persons from different professions learn and work together (52). Therefore, FINCA takes the individual learning prerequisites of two or more persons from health care professions A and B into account. IPE is about mutual recognition of roles and responsibilities. These vary from profession to profession as well as within professions that specialize. When collaborating interprofessionally, it is important to understand who you are working with and how best to use the skills of each profession and individual for successful collaborative problem-solving. Less optimal practice results from not having this understanding (1).

In the context of teaching and learning, knowledge is understood as the mental representation of information (53). Under *cognitive professional abilities*, FINCA includes *conceptual* and *procedural knowledge* as well as *collaborative knowledge* (54). Under *motivational and affective factors*, FINCA includes *professional identity* as well as *interest*, *engagement*, and *attitude toward the subject matter.*

A comprehensive research program on IPE and its instructional facilitation must address the question of how and to what extent individual learning prerequisites affect the outcomes of IPE and IPC. The benefit of clarifying the relationship between instructional effects and pre-existing individual differences among learners is obvious. Systematically incorporating individual learning prerequisites addresses the question for whom particular instructional designs are likely to be effective. Scientific insights into the moderating effects of individual learning prerequisites can help make interprofessional learning environments more effective and could serve as the basis for individualized and adaptive facilitator support measures that address the learning needs of each professional. This could involve the aforementioned use of simulations that address learning outcomes relevant to learners of all health professions involved (55, 56).

In the following, we propose cognitive, affective, and personality related moderators which can serve as a starting point for more systematic research on how learning prerequisites affect the processes and outcomes of interprofessional patient care and learning with and without additional instructional support.

#### 3.2.1. Cognitive professional abilities

The basis of knowledge acquisition lies in the formation of concepts and contexts in a specific learning area, such as medicine or care. In contrast to this *conceptual knowledge*, which can also be referred to as factual knowledge, *procedural knowledge* focuses on the procedure and steps to be followed in clinical problem-solving. Procedural knowledge includes both strategic knowledge (about typical problem-solving strategies) and conditional knowledge (about conditions of application of conceptual and strategic knowledge) (54, 57). Besides individual cognitive structures of learners, *collaborative knowledge* is an essential element of cognitive professional abilities in FINCA. Collaborative knowledge comprises cognitive activities (e.g., explaining, questioning, summarizing), metacognitive activities (e.g., observing, regulating, formulating arguments), as well as social activities (e.g., taking turns, listening) (19). Although collaborative practice is commonplace in clinical settings, there has been little empirical research on how to analyze and promote the skills required for it (12).

Another view on the professional knowledge base in health care differentiates between biomedical and clinical knowledge (58, 59). Biomedical knowledge includes knowledge about physiological, pathological, as well as psychosocial elements. Clinical knowledge, on the other hand, includes symptoms, symptom patterns and clinical pictures, typical disease courses, as well as suitable therapeutic procedures. While the biopsychosocial model of medicine by Engel (60) is still relevant, there are calls for expanding this model toward a health care system perspective (61). From an interprofessional perspective, such an expansion should comprise perspectives and values of all professions contributing to health and patient care. One outcome of IPE could be, for instance, that health professionals better understand the importance of social and cultural factors for health and illness from diverse professional perspectives and recognise their significance in the care process [*cf.* (62)].

#### 3.2.2. Motivational and affective factors

FINCA systematically addresses individual learning prerequisites. This includes *motivational and affective factors* such as *interest*, *engagement*, and *attitude toward the subject matter*. The framework also considers the influence of the development of *professional identity* in the respective professions involved in IPC. At this point, however, it should be pointed out that there is currently no uniform definition of professional identity within an interprofessional context (63, 64). To our knowledge, there has been no systematic research examining potential moderating effects of motivational and affective factors on the development of interprofessional activities in IPE.

### 3.3. Noticing

In teacher education research, *noticing* has been described as a process that lets teachers’ pay attention to significant events within teaching and learning in the classroom (29, 30). Applied to the clinical context, we suggest that noticing can analogously be understood as a psychological process leading to interprofessional interaction and the corresponding collaborative activities.

We propose that noticing occurs at the beginning of any interprofessional interaction or collaboration when there is a realization that collaboration with other health care professions must be initiated to jointly address specific needs of a patient. A distinction can be made between spontaneous noticing, when an unexpected situation requires IPC (e.g., decision to treat a wound with a vacuum pump), and ritualized IPC where noticing happens implicitly (e.g., interprofessional surgical ward rounding). In FINCA, noticing marks the transition to observable collaborative activities (see Figure 1). The communicative part of noticing itself might already be observable and thus amenable to an assessment.

Linking the theoretical elements described earlier, one could hypothesize that noticing leads to an activation of illness scripts and the complementary internal collaboration scripts among the individuals involved. The interaction of individual participants in a given situation thus depends on their memory structures related to their respective memories of a specific social situation (e.g., patient handover). In our view, these memory structures can be conceptualized as internal collaboration scripts that individuals can draw on depending on the situation (19, 25).

### 3.4. Observable collaborative activities

At the heart of FINCA are observable collaborative activities that can be used to assess individuals’ cognitive and social skills. Liu et al. (20) postulate that collaborative learning scenarios can promote greater integration of knowledge and thus lead to better learner performance. To this end, they propose a conceptual model that includes a matrix of individual cognitive and social skills involved in collaborative problem-solving. This model can also serve as a basis for assessing an individual’s collaborative problem-solving skills. Liu’s assumptions are based on research in computer-supported collaborative learning (65) and also draw on the PISA 2015 Collaborative Problem Solving Framework (66). The conceptual model by Liu and colleagues also considers individual cognitive prerequisites and assigns the following key social skills to them: *information sharing*, *negotiating*, *regulating*, and *maintaining communication*. In order to incorporate clinical practice, FINCA further adds the more practice-oriented activity *executing interprofessional activities*.

#### 3.4.1. Information sharing and grounding

The activity *information sharing* captures how individual group members contribute different ideas to a common conversation (20) or point to relevant resources that help to solve a problem that requires IPC. However, it is important that not only information is shared, but that communication partners also strive for mutual understanding. Clark and Brennan (67) refer to this process as *grounding*.

#### 3.4.2. Negotiating

In the context of IPC, the term *negotiating* is often used in the sense of negotiated order theory [e.g., (21, 68)]. In FINCA, negotiating refers specifically to conversations about the team’s collaborative knowledge construction by comparing alternative ideas and information resources, presenting evidence, and justifying an argument. Subcategories of this activity include asking for clarification, elaborating/reformulating a collaboration partners’ ideas, identifying knowledge gaps, and revising/reformulating one’s own ideas (20).

#### 3.4.3. Regulating

*Regulating* problem-solving activities refers to conversations about clarifying objectives, monitoring, evaluating, and confirming team understanding of problem-solving. This category focuses on the collaborative regulation aspect of team conversations. It includes subcategories such as identifying aims, evaluating teamwork, and checking mutual understanding regarding aims that were jointly agreed upon (20). In clinical practice, this would occur, for example, when an interprofessional team agrees on a joint management plan for a patient.

#### 3.4.4. Executing interprofessional activities

The observable collaborative activities mentioned previously refer to cognitive and communicative skills. They prepare for *executing interprofessional activities* that can be assessed on the grounds of clinical standards, guidelines, and patient safety requirements. Instruments for the assessment of interprofessional team collaboration have recently been developed and evaluated [e.g., (69–71)].

#### 3.4.5. Maintaining communication

*Maintaining communication* includes all activities related to the conversational climate. This encompasses all conducive activities that enable efficient and effective dyadic communication between a person A and a person B (see Figure 1) or within interprofessional teams. This also includes avoiding professionally irrelevant social communication (20).

To exemplify the proposed observable collaborative activities we describe a realistic clinical scenario in Table 1. It is important to note that the procedures can be repeated several times and are by no means a linear process. In our example, a nurse and a physician on a ward must jointly decide whether to place a permanent bladder catheter in a patient based on clinical data and observations.

**Table 1:** Placement of a catheter as an illustration of observable collaborative activities.

Clinical scenario: The patient is Mr. Anton Smith, 88 years old, with a diagnosis of congestive heart failure. Mr. Smith was admitted yesterday after a fall at home with a fracture of the neck of the femur. The ward physician orders a 24-h fluid balance for the patient.	
Activity	Description
Noticing	The nurse seeks discussion with the ward physician because she considers fluid balancing with the urine bottle Mr. Smith uses to be unfeasible.
Maintaining communication	Before the conversation begins, the ward physician asks about the current mood in the nursing team as a nurse is sick today. That is why the team is under high time pressure.
Information sharing and grounding	The nurse reports that Mr. Smith is unable to perform his intimate toilet independently and has difficulty urinating as he is partially incontinent. The ward physician informs the nurse about Mr. Smith’s prostate adenoma and a worsening of the lung congestion in the chest X-ray.
Negotiating	The nurse suggests placing a transurethral indwelling bladder catheter for accurate fluid balancing due to the severity of Mr. Smith’s clinical condition. The ward physician agrees and additionally suggests daily weight measurement.
Regulating	After a joint consideration of the benefits for the patient, the ward physician and the nurse jointly decide to insert a transurethral permanent bladder catheter. Because of the prostate adenoma, they decide to place the catheter together under ultrasound control.
Executing interprofessional activities	The nurse informs Mr. Smith about the indication to place a permanent catheter and obtains his consent. She informs the ward physician and prepares all necessary materials for the procedure. She positions Mr. Smith flat on his back and the bladder catheter is placed under ultrasound control. The ward physician sounds and instructs. The nurse inserts the permanent bladder catheter under sterile conditions into the bladder and attaches the urine bag. She performs intimate care and repositions Mr. Smith together with the ward physician. The nurse then instructs Mr. Smith on how to use the indwelling urinary catheter.

### 3.5. Action-modifying context factors

FINCA acknowledges *action-modifying context factors* as potentially significant moderators of interprofessional collaborative activities. The literature suggests a variety of such factors that may influence the effectiveness and efficiency of interprofessional collaborative practice [e.g., (5, 72, 73)]. However, the evidence base regarding their impact is not sufficient (74). Mulvale et al. (75) identified a number of studies that measured correlations between collaborative processes in interprofessional practice and structural and process factors. Thus, FINCA includes action-modifying context factors which can be divided into *team factors (micro-level)*, *organizational factors (meso-level)*, and *system factors (macro-level)*. At each level, we focus on the contextual and process-related factors that we propose to be associated with interprofessional collaborative activities. While not all of the relevant factors on the respective levels can be influenced to the same extent, we believe they are important to a framework that aims to comprehensively address IPC in health education and care.

#### 3.5.1. Micro-level

Under the micro-level, we subsume formal and informal factors that can influence a team, such as *team size and role clarity*, *team composition*, *trust* between team members, as well as the team’s *experience* with IPC. We also consider *leadership and hierarchies*, as well as *communication culture* within teams as important (76, 77).

#### 3.5.2. Meso-level

The meso-level comprises *institutional and economic factors*, *infrastructure and environment*, and *organizational culture*. It should be noted that context factors on the meso-level are typically beyond the control of individual health professionals.

#### 3.5.3. Macro-level

The macro-level includes the *health care system and its regulations*, which should be considered on both the educational planning and the organizational side. The context factors on the macro-level are even harder to change in order to improve IPE and IPC.

### 3.6. Outcomes of IPE and IPC

Following Heitzmann et al. (18) and Liu et al. (20), FINCA aims to capture and assess outcomes of IPE and IPC, both of which are complex and multifaceted constructs. In addition to assessing entire teams and their performance, there is an urgent need in research to develop stable and robust outcome criteria to demonstrate a causal relationship between IPE, IPC, and overall health care system outcomes.

#### 3.6.1. Results of IPC

Bodenheimer and Sinsky’s (78) Quadruple Aim concept is now widely recognized as a compass for optimizing health care delivery and is being further developed as a standard assessment criterion for IPC [e.g., (79)]. Following this concept, we have included the following promising criteria for assessing results of IPC in FINCA: (1) *Accuracy* (improved patient outcomes); (2) *Improved patient experience*; (3) *Improved clinical experience of the interprofessional team*; (4) *Efficiency* (reduced costs).

#### 3.6.2. Individual outcomes of IPE and IPC

On top of the prevalent profession-specific assessment instruments for conceptual and procedural knowledge (*improved cognitive professional abilities*), instruments are needed that allow for the assessment of interprofessional collaborative activities [e.g., (71)]. According to the current IPE literature (80), individual performance should be assessed separately from team performance when evaluating collaboration in health care (*improved collaborative activities*). One possibility in this respect is offered by the concept of entrustable professional activities (EPAs). Simply put, this is about detailed authentic descriptions of clinical activities that health care trainees can be entrusted with (81). Recently, transdisciplinary EPAs have been conceptualized to be used for multiple professions (82).

## 4. Discussion

The present conceptual framework has been developed with scientific consideration of empirical data under various theoretical references. In our view, FINCA adequately reflects the process of IPC in a clinical context building on established theoretical foundations – it operationalizes contextual, person-related, process-related, and outcome-related variables (23) to capture what we postulate to be the observable part of IPC. In this way, the framework provides the basis for analysis and empirical testing of the components and variables described, as well as their interactions across different studies, educational interventions, and action-modifying contexts (micro-, meso-, and macro-level). FINCA further provides the basis for fostering the teaching and learning of interprofessional problem-solving skills across different health care settings.

In addition, FINCA may support faculty and curriculum developers to systematize the implementation and improvement of interprofessional teaching and learning opportunities [*cf.* (83)]. From a practical perspective, FINCA can help to better align curricula for different health professions in the future.

The proposed framework does not claim to be a theory or model yet – as yet, this is a qualitative synthesis of published literature, the empirical confirmation of which is pending. We invite readers to discuss modifications, additions, innovations, or other perspectives with us. Perspectives from all professional groups involved in health care are explicitly welcome. In principle, we also see potential for transferability of the framework to other domains where different professions collaborate.

## Data availability statement

The original contributions presented in the study are included in the article/supplementary material, further inquiries can be directed to the corresponding author.

## Author contributions

MW, JZ, BW, FF and MF jointly developed the first version of the framework model. MW and JZ wrote the first draft of the manuscript with support from BW. This draft was commented on and expanded by FF, JT and MF. The manuscript was eventually revised by MW, JZ, JT and MF. All authors contributed to the article and approved the submitted version.
